# The Effect of Single Pyramidal Neuron Firing Within Layer 2/3 and Layer 4 in Mouse V1

**DOI:** 10.3389/fncir.2018.00029

**Published:** 2018-04-16

**Authors:** Jochen F. Meyer, Peyman Golshani, Stelios M. Smirnakis

**Affiliations:** ^1^Department of Neuroscience, Baylor College of Medicine, Houston, TX, United States; ^2^Department of Neurology, University of California, Los Angeles, Los Angeles, CA, United States; ^3^Department of Neurology, Brigham and Women's Hospital and Jamaica Plain Veteran's Administration Hospital, Harvard Medical School, Harvard University, Boston, MA, United States

**Keywords:** two-photon microscopy, calcium imaging, electrical stimulation, mouse V1, cortex, functional connectivity, *in vivo* patch-clamp recording

## Abstract

The influence of cortical cell spiking activity on nearby cells has been studied extensively *in vitro*. Less is known, however, about the impact of single cell firing on local cortical networks *in vivo*. In a pioneering study, Kwan and Dan (Kwan and Dan, 2012) reported that in mouse layer 2/3 (L2/3), *under anesthesia*, stimulating a single pyramidal cell recruits ~2.1% of neighboring units. Here we employ two-photon calcium imaging in layer 2/3 of mouse V1, in conjunction with single-cell patch clamp stimulation in layer 2/3 or layer 4, to probe, in *both the awake and lightly anesthetized states*, how (i) activating single L2/3 pyramidal neurons recruits neighboring units within L2/3 and from layer 4 (L4) to L2/3, and whether (ii) activating single pyramidal neurons changes population activity in local circuit. To do this, it was essential to develop an algorithm capable of quantifying how sensitive the calcium signal is at detecting effectively recruited units (“followers”). This algorithm allowed us to estimate the chance of detecting a follower as a function of the probability that an epoch of stimulation elicits one extra action potential (AP) in the follower cell. Using this approach, we found only a small fraction (<0.75%) of L2/3 cells to be significantly activated within a radius of ~200 μm from a stimulated neighboring L2/3 pyramidal cell. This fraction did not change significantly in the awake vs. the lightly anesthetized state, nor when stimulating L2/3 vs. underlying L4 pyramidal neurons. These numbers are in general agreement with, though lower than, the percentage of neighboring cells (2.1% pyramidal cells and interneurons combined) reported by Kwan and Dan to be activated upon stimulating single L2/3 pyramidal neurons under anesthesia (Kwan and Dan, 2012). Interestingly, despite the small number of individual units found to be reliably driven, we did observe a modest but significant elevation in aggregate population responses compared to sham stimulation. This underscores the distributed impact that single cell stimulation has on neighboring microcircuit responses, revealing only a small minority of relatively strongly connected partners.

**One sentence summary:**

Patch-clamp stimulation in conjunction with 2-photon imaging shows that activating single layer-2/3 or layer-4 pyramidal neurons produces few (<1% of local units) reliable single-cell followers in L2/3 of mouse area V1, either under light anesthesia or in quiet wakefulness: instead, single cell stimulation was found to elevate aggregate population activity in a weak but highly distributed fashion.

**Summary of the findings:**

1. We implemented a new statistical approach that can identify follower cells activated in at least ~40% of all trials more sensitively and specifically than prior methods.
2. *In vivo* single-cell activation through patch-clamp recordings causes reliable activation in <1% of neurons (“reliable follower cells,” as described in #1) within a radius of ~200 μm in layer 2/3 of mouse V1.
3. Single pyramidal neuron stimulation at layer 4 did not yield more followers in L2/3, compared to intralaminar L2/3 stimulation.
4. There was no difference in the number of followers seen after single pyramidal cell stimulation in light anesthesia vs. quiet wakefulness.
5. Although single cell stimulation drives few individual units reliably, it does cause a modest but significant elevation in aggregate population responses compared to sham stimulation, within an area of a ~200 μm radius.

## Introduction

It is important to understand how a single neuron's spiking activity influences nearby cortical circuit function. Using a simple network model, Shadlen and Newsome (1998) estimated that, absent inhibition, a neuron can produce an AP in response to 10–40 input spikes with 10–20 ms interspike-intervals (ISI). This suggests that physiological presynaptic activity in just a single cell is potentially capable of driving its postsynaptic partners, if the cell fires at high rates.

This has been the subject of recent investigation, with partially conflicting results. It has been argued that several dozen neurons need to be simultaneously active to drive behavioral tasks in the mouse (Huber et al., 2008), or to elicit postsynaptic spiking in guinea pig primary visual cortex (V1) slices (Sáez and Friedlander, 2009). On the other hand, other studies suggest that single cell firing can influence local and global network activity and even behavior significantly. For example, *single* unit firing has been reported to (i) increase the firing rate of postsynaptic targets (London et al., 2010), (ii) stabilize network activity sparseness (Ikegaya et al., 2013), (iii) elicit whisker movements (Brecht et al., 2004), (iv) switch between global up-and down states (Li et al., 2009), and (v) elicit behavioral somatosensory responses (Houweling and Brecht, 2008). These studies suggest that single unit activity can influence neural network state (Li et al., 2009) and even animal behavior (Houweling and Brecht, 2008).

Less is known about the effect that the activation of a single neuron has on its local circuit environment. The target units, or “followers,” together with the pre-synaptic neuron, which recruits them to fire, constitute a basic module of cortical computation. This module transforms the information represented by the firing pattern of a single unit into a distributed pattern of activity in specific follower neurons. Here we begin to probe the basic rules of this transformation in the visual system, taking into account brain state and the cortical layer of the parent neuron. It is important to understand how single unit activity influences neighboring neuron activity under physiological conditions, *in vivo*, since *in vitro* studies inevitably disturb the cortical circuit, via the loss of mid-and long-range axonal connections (Stepanyants et al., 2009).

Kwan and Dan recently used single-cell stimulation in conjunction with two-photon calcium imaging to show that ~1.7% of neighboring pyramidal cells (“followers”) could be driven by burst firing of a patched pyramidal neuron (Kwan and Dan, 2012) in L2/3 of mouse area V1. Since this pioneering work was performed under anesthesia it remains unclear whether it applies to the awake brain state. Activity patterns in sensory cortex differ significantly in wakefulness vs. under anesthesia. In particular, inhibition in L2/3 of mouse V1 is weaker under anesthesia, whereas in the awake state it significantly restricts both spatial and temporal patterns of activity (Haider et al., 2013). Furthermore, it has been suggested that pyramidal cell firing may be propagated with different efficiency across vs. within cortical layers (Beltramo et al., 2013). Recent work *in vitro* suggests that excitatory neurons form sparse but strongly connected sub-networks (Yoshimura et al., 2005), which display stronger excitatory drive from L4 to L2/3 vs. within L2/3 itself (Xu et al., 2016). It remains unclear however, how these sub-networks behave *in vivo*.

To investigate this question, we probed the ability of a single pyramidal unit to influence the action potential output of another (“effective connectivity”) *in vivo*, by electrically stimulating pyramidal neurons in L2/3 or L4 via single-cell patch-clamp, while recording neuronal activity in L2/3 using two-photon calcium imaging. For this, it was important to develop a new algorithm that is both sensitive and specific for identifying activated or inhibited “follower” cells that exhibit strong effective connectivity to the stimulated unit.

## Methods

### Breeding

Viaat-Cre mice expressing Cre in >98% of GABAergic interneurons (Chao et al., 2010) were back-crossed with C57/BL6 mice and then crossed with Ai9 (C57/BL6) mice (containing a stop-floxed tdTomato gene). The offspring expressed tdTomato, a red fluorescent protein, in ~98% of all interneurons. Analogously, for select experiments we used the F1 offspring of PV-Cre (C57/BL6) x Ai9 crosses which expressed tdTomato in all parvalbumin-positive (PV+) interneurons, and DLX5/6-Cre x Ai9 crosses expressing tdTomato in all interneurons originating from the medial ganglionic eminence, which includes most of the PV+ and somatostatin-positive (SOM+) GABAergic interneurons. For 4 of the L4 stimulation experiments, Scnn1a-cre (C57/BL6) x Ai9 mice were used. These animals express tdTomato selectively in pyramidal cells in L4.

### Surgery

All procedures described here were carried out according to animal welfare guidelines. Anesthesia was induced and maintained with 1.5% isoflurane using a stereotactical stage for mice (Kopf Instruments). Eyes were protected with a thin layer of polydimethylsiloxane (30,000 cst, Sigma-Aldrich) for the duration of the surgery. A custom-made titanium head plate was attached with dental acrylic (Lang Dental), mixed with charcoal powder for light shielding. A 3-mm wide, circular craniotomy was made, centered over the middle of the monocular region of V1 (2.5 mm lateral of the midline, 1.2 mm anterior of the lambda suture). A 5-mm diameter, 0.16 mm thick round cover glass with a hole for pipette access was placed on the brain after the bone surrounding the hole was flattened carefully in order to avoid a gap between the glass and the brain, which could lead to pinching of blood vessels on the edge of the bone. An Ag/AgCl reference wire was either implanted in the cerebellum (~2 mm posterior of the lambdoid suture contralateral to the craniotomy), or immersed in the saline bath above the brain during the recording.

### Dye loading and imaging

Fifty micrograms Oregon Green 488 BAPTA-1 AM (Invitrogen) was dissolved in 4 μL heated (40°C) dimethyl sulfoxide (DMSO) with 10% Pluronic acid F-127 (Invitrogen), vortexed for 20 min, and diluted in 40 μL 0.9% NaCl solution containing 10 μM Alexa-594 for experiments with tdTomato-labeled interneurons, and 10 μM Sulforhodamine 101 (Nimmerjahn et al., 2004) for selective astrocyte-labeling in other experiments. The filtered solution was injected under two-photon guidance using a glass pipette with ~3 μm tip diameter. The pipette was advanced slowly using a motorized manipulator (Sutter MP-265), and 2–5 PSI of positive pressure were applied carefully with a 20-mL syringe, such that a sphere with diameter ~300 μm would be filled within 2 min. Typically, several overlapping injections were made at depths of 200, 300, and 400 μm and 2 or 3 different locations, to maximize the stained volume. After pressure was set to zero, the pipette was removed carefully. Recording commenced 1 h following the dye injection, allowing time for the cells to take up the injected Oregon-green BAPTA (OGB). Two-photon imaging was performed using a modified Prairie Ultima IV two-photon laser scanning microscope (Prairie Technologies, Middleton, WI) with a beam expander, fed by a Chameleon Ultra femtosecond laser (Coherent, Santa Clara, CA). 40x, 0.8 NA (Nikon), 20x, 1.0 NA (Olympus), or 25x 1.1 NA (Nikon) lenses were used to scan fields of view (FOVs) containing between 20 and 75 neurons at 5–15 Hz. Depending on objective used and imaging depth, the laser power was kept between 10 mW at the surface and 50 mW at depths below 250 μm, at wavelength of 840 nm (when the patch pipette was filled with Alexa 594) or 890 nm (when filled with dextran).

### Patch clamp recording and stimulation

**Figure 1:**
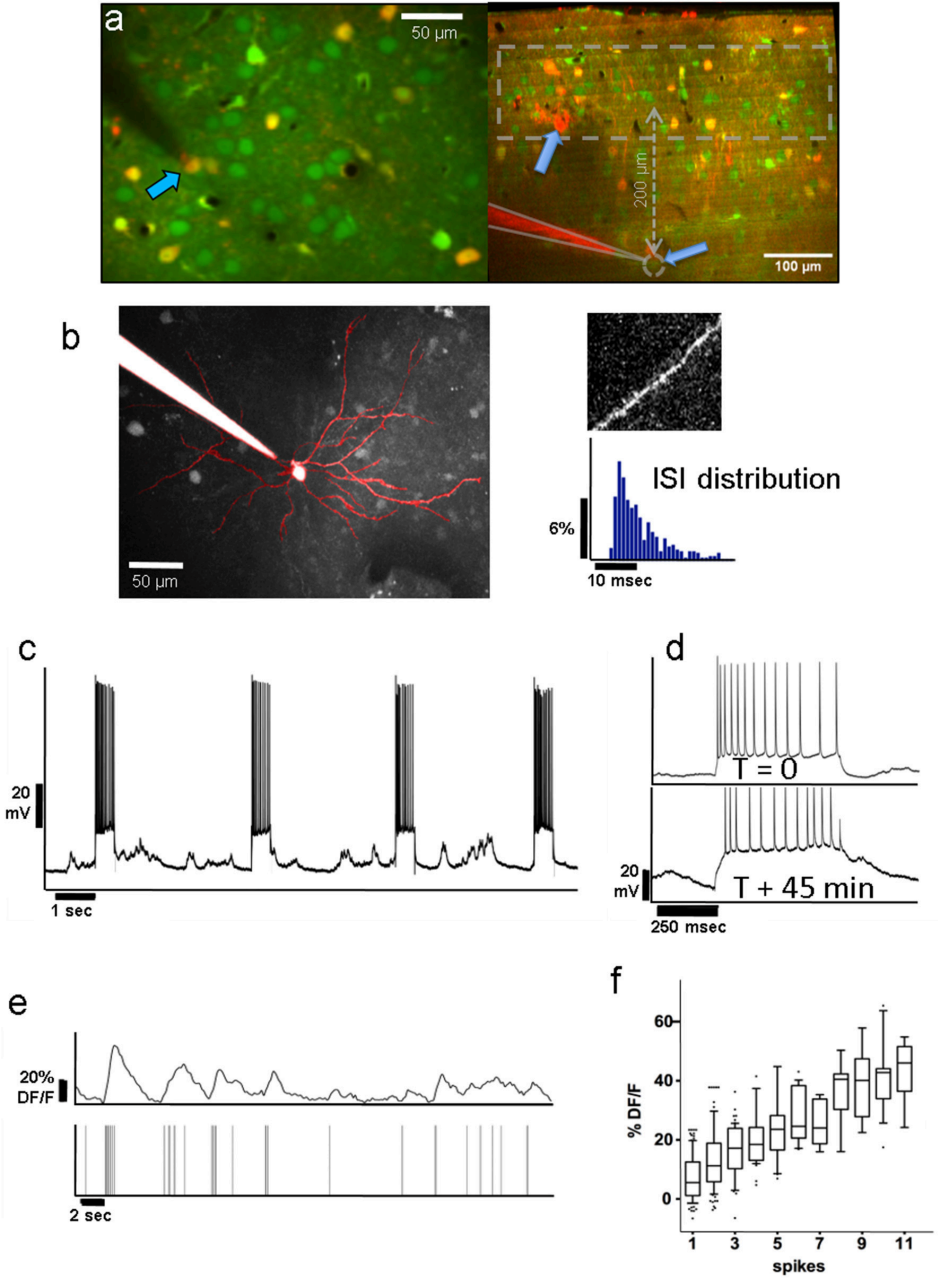
**(a)** Left: Group of layer-2 OGB-labeled neurons in a Viaat-Cre x Ai9 mouse whose interneurons are labeled with tdTomato (yellow); pyramidal neurons appear green. The whole-cell patched cell (arrow) appears orange because it is filled with both OGB and Alexa 594 from the pipette solution. This allowed us to confirm the identity of the neurons we stimulated in whole-cell patch. The scale bar is 50 μm. Right: Coronal view of L2/3 and L4 of OGB-labeled area V1 showing the tip of a patch pipette after recording from a L4 neuron ~370 μm below the pia in cell-attached mode. Interneurons are labeled red. The dashed area represents the range of depths we recorded the responses of L2/3 neurons from. **(b)** Average projection of the top 130 μm of a z-stack containing a whole-cell patched L2/3 neuron passively filled with Fluoro-Ruby dextran. It displays the typical apical dendrite pattern of an L2/3 pyramidal cell projected in the x-y plane, including spines seen on its superficial dendritic segments (see top right). We compared the firing patterns and spike wave shapes of all patched neurons with those of confirmed pyramidal cells to ensure that we were only stimulating pyramidal units. The bottom right panel shows a typical distribution of ISIs from one of the stimulated pyramidal cells. **(c)** Voltage trace of the neuron in whole-cell patch shown in panel **(a)** during electrical stimulation. Note the high reliability with which APs are elicited during electrical stimulation over the course of ~45 min of stimulation (see panel **d**). **(d)** Voltage traces during stimulation of the same neuron at the beginning of a recording (top), and 45 min later (bottom), demonstrating that patched neurons can be stimulated reliably over long periods of time. The resistance drifted somewhat over time, but the number of elicited spikes remained similar. Stimulated neurons always fired multiple spikes (12–15 on average) per stimulation epoch (see section Methods/Results). Note that the percentage of followers per stimulated cell did not depend on the average number of elicited spikes per recording (see Supplementary Results). **(e)** Top Trace: Typical Calcium activity spontaneously generated by the L2/3 neuron from **(d)**. Bottom Trace: Timestamps of recorded APs. Note the close correspondence between the calcium signal and underlying APs. **(f)** On average, the ΔF/F amplitude of the calcium signal (y-axis) corresponds well with the number of APs (x-axis). The upper and lower box bounds depict the 25th and 75th percentile, respectively, while whiskers extend from the 5th to the 95th percentile.

Whole-cell and loose-patch recordings were obtained with a Heka EPC-10 USB amplifier in current-clamp mode using standard techniques (Margrie et al., 2003). Briefly, 6–8 MΩ glass pipettes filled with intracellular solution (in mM: 105 K-gluconate, 30 KCl, 10 HEPES, 10 phosphocreatine, 4 ATPMg, and 0.3 GTP), adjusted to 290 mosm and pH 7.3 with KOH (Golshani et al., 2009) and containing 10 μM Alexa-594 or tetramethylrhodamine dextran (Invitrogen), were advanced under visual two-photon guidance, initially with ~100 mbar pressure dropping to ~40 mbar when ~50 μm below the dura. When approaching a cell, pressure was further reduced to ~20 mbar. Once resistance increased to ~200% of the initial value, laser scanning was stopped and up to 200 mbar negative pressure was applied, until the resistance increased to 200 MΩ. When successful, a multiple GΩ seal typically formed within 2 min. The pipette was then retracted carefully by a few μm to avoid penetrating the interior of the cell, and ~200 ms pulses of negative pressure with increasing strength were applied via a Picospritzer with a vacuum module until a patch of cell membrane was broken. Fast pipette capacitance was neutralized before break-in, and slow capacitance afterwards. Before electrical stimulation commenced, several minutes of spontaneous activity were recorded to ensure the patch was of good quality and that normal spiking activity did not deteriorate quickly, which would indicate a poor seal or cell damage. Once the whole cell patch was deemed stable and the cell healthy, 200–2,000 ms current pulses were applied at increasing amplitudes, starting at 100 pA to find the firing threshold of each cell. Typically, 200–300 pA (max. 400 pA for whole-cell stimulations and 1,000 pA for cell-attached stimulations in order to minimize cell damage over time) were required to elicit burst spiking reliably over long periods of time, but the duration and amplitude were adjusted for each recording such that on average 12–15 spikes were elicited per pulse. On average, the current pulses were 505 ± 67 ms (sem, in L2/3 anesthetized), 529 ± 29 ms (sem, L2/3 awake), or 409 ± 53 ms (sem, L4 awake) long, and elicited spiking at average firing rates of 24–37 Hz. Between electrical pulses, the cells were hyperpolarized to avoid spontaneous spikes outside the stimulation periods (this required injecting −50 to −200 pA in whole-cell configuration and up to −900 pA in cell-attached mode, depending on the spontaneous excitatory drive of each individual cell). In 11 out of 47 recordings, where a good seal, but not a GΩ seal, was achieved (i.e., cell attached mode), neurons were stimulated with up to 1,000 pA to elicit spiking, and by −900 pA to suppress firing. Recordings where neither a stable GΩ seal nor a stable MΩ seal could be established were not analyzed. There were only 2 L2/3 anesthetized experiments with cell-attached stimulation, and 3 in awake mice, making it impossible to judge whether the type of patch configuration had an influence on the stimulation. However, in L4 stimulation experiments, there were 6 cell-attached recordings, which did not have different numbers of followers than the whole-cell recordings (*p* = 0.95). We targeted pyramidal cells either in L2/3 (between 100 and 250 μm below the pia), or in L4 (between 320 and 370 μm below the pia, according to Niell and Stryker, 2008). All stimulated L4 cells were located directly below the imaged field of L2/3 cells, well within the bounds of their FOV (Figure 1a). For all experiments, we were confident that we stimulated pyramidal cells based on morphology, accommodating spike trains in response to current pulses, and the genetic labeling of inhibitory cell types in a subset of animals.

### Data analysis

**Figure 2:**
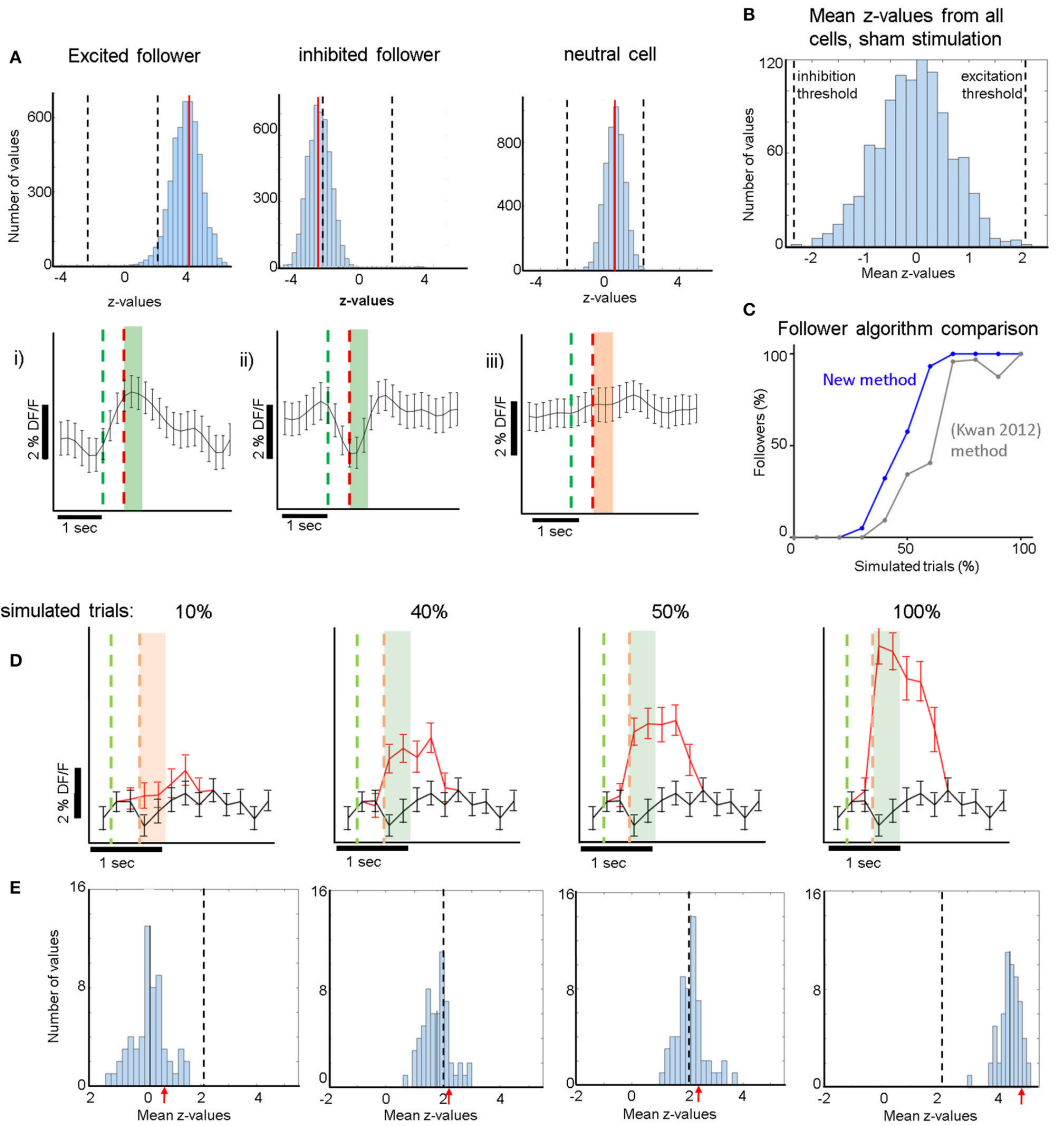
**(A)** Top Row: **(i)**: Cyan: histogram of the distribution of *z*-values derived from comparisons between the real responses to electrical stimulation and 5,000 iterations of randomly shuffled responses (see section Methods). Red Line: mean of the distribution. Dashed Lines: Thresholds for the *z*-value mean, above which excited followers are identified, and below which inhibited followers are identified. This is derived from the sham stimulation experiments, to yield high specificity, i.e., no sham followers **(B)**. **(ii)** Analogous to **(i)** for an inhibited follower cell. The red line (mean of the distribution) now lies below the lower threshold, below which inhibited followers are identified. **(iii)** Analogous to **(i,ii)** for a typical non-follower (neutral) cell. Note that mean *z*-value of the cyan histogram did not cross either threshold. Bottom Row: Corresponding average responses (±sem) of the excited **(i)**, inhibited **(ii)**, and neutral **(iii)** neurons shown in the top row. For **(i,ii)** trials which contributed to significance (see section Methods) were averaged. Green line: stimulation onset. Red line: stimulation end. Green shaded areas **(i,ii)** indicate which frames were averaged to compute the response (400 ms after stimulus offset). Orange shaded area in **(iii)** analogous for a non-follower. **(B)** Distribution of all *z*-value means from all 1,069 cells that received sham stimulation. *z*-value means never exceeded 2.1 (“excitatory-follower threshold”) or fell below −2.3 (“inhibitory-follower threshold”). **(C)** Simulation shows that our follower algorithm was more sensitive than a previous method by Kwan and Dan (2012). It identified more neurons correctly as followers at almost all levels of simulation, identifying essentially all followers that have an additional spike elicited >60% of the time upon stimulation. The sensitivity drops sharply below that, so that the algorithm identifies only ~50% of followers that have one additional spike elicited ~40% of the time upon stimulation. **(D)** Simulation of activity modulation when a calcium signal equivalent to 1 AP was added to 10, 40, 50, or 100% of all trials. Red trace: average ΔF/F signal (±sem) of an example neuron after addition of 1-AP calcium transients. Black trace: control with no added activity. Our algorithm identified this neuron as a follower when at least 40% of all trials received a simulated extra AP. The red and green dashed lines represent the periods where the single cell would have been stimulated electrically (analogous to **A**). Note that here electrical stimulation does not occur but instead an extra action potential is inserted at stimulus offset. Note that because the frame rate was lower than the calcium signal rise time, it can appear as though the calcium signal starts to rise within a frame before the time stamp of the AP, due to alignment jitter. The light green and light orange shaded areas indicate the frames that were averaged to calculate the simulated stimulus response (400 ms). **(E)** The blue histograms at the bottom represent the mean *z*-values across all 75 cells (one FOV) receiving simulated 1-AP calcium signals in 10, 40, 50, or 100% of all trials. Here, we combined data from 2 spontaneous activity recordings that had 190 simulated trials, which was the typical number in our experiments. This shows that our algorithm identifies ~50% of neurons as followers when at least 40% of the trials receive one extra AP. Red arrows indicate the z-scores corresponding to the simulated panels shown in **(D)**. Dashed Line: Threshold for excited follower identification.

Custom written Matlab algorithms were used for motion correction (FFT-cross correlation of all image frames with a reference frame) and all subsequent analysis steps (see Figure 2). ImageJ was used to manually select cell outlines for generation of the calcium activity traces. Pixels within each region of interest (ROI) were averaged for each image frame, and fluorescence values were converted into ΔF/F percent change using the equation ΔF/F = F_s_/F_b_ − 1, where F_s_ is the fluorescence signal 0–400 ms after the end of each electric stimulus (see Figure 2), and F_b_ is the local baseline amplitude 600–0 ms before the onset of a stimulus. We also implemented a custom statistical approach for identifying “follower” cells (see section Results, Figure 2).

Due to the inherent variability of the calcium signal in each neuron, it was important to develop a sensitive but specific algorithm to identify statistically significant “follower” cells. Each cell's calcium trace was pre-processed as described above and then processed further in 3 steps: The peak of the calcium activity profile elicited during stimulation trials was measured in the patched cells and was found to be ~200 ms following the end of stimulation. Average single-spike calcium transients were found to have a width at half maximum of 400 ± 200 ms. We therefore computed the response for each trial by averaging 400 ms of the ΔF/F trace starting right at the offset of the stimulation pulse and normalizing by the 600 ms baseline activity measured before the onset of the pulse, thereby obtaining ΔF/F. These values, i.e., the responses r(t) for each trial t, were combined in a distribution **R** of real stimulation responses. In the second step, the calcium trace of each cell was shifted 5,000 times in a circular fashion by a random number of frames (avoiding shifts corresponding to 1 s around another stimulus pulse, i.e., the average calcium transient duration corresponding to 1 AP), creating 5,000 distributions of null responses **R**_N_ (N:null). In the third step, we first computed 5,000 *z*-values (using the Wilcoxon ranksum test) between the real distribution **R** and the 5,000 iterations of **R**_N_. Then, we calculated the mean of that distribution of *z*-values for each cell and compared it to a threshold that was derived from all cells in the 19 FOVs that underwent sham stimulation (*n* = 1,069 cells). See Supplementary Figure 2 for a visual outline of this process. As shown in Figure 2B, the mean of 5,000 *z*-values never lies above 2.1, or below −2.3, if a sham stimulus is applied to the patched cell eliciting no APs. We therefore conservatively considered a cell near a patched neuron receiving electrical stimulation to be an excited follower when its mean *z*-value exceeded 2.1, and an inhibited follower when it lay below −2.3.

**Figure 3:**
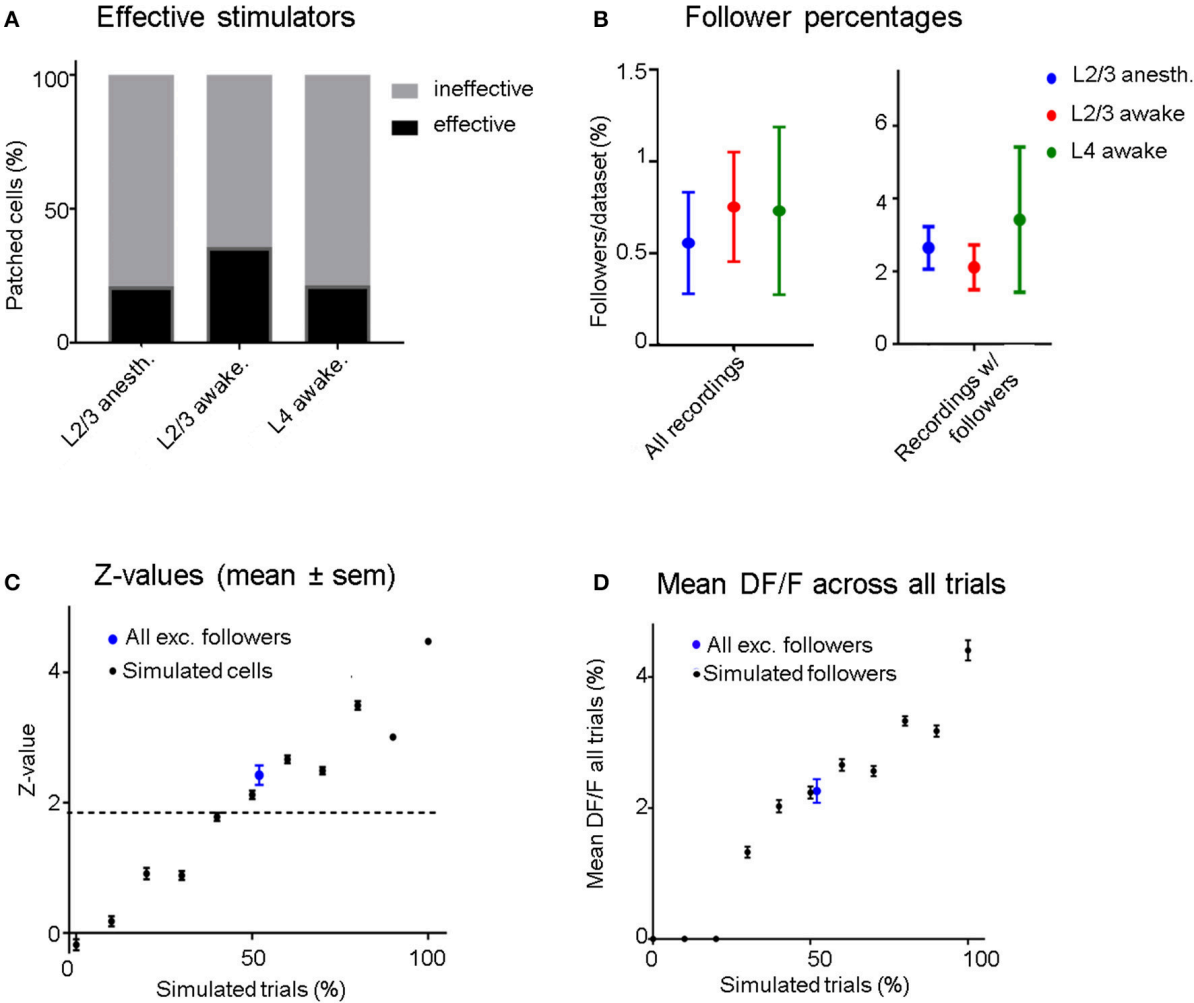
**(A)** Four out of nineteen (21%) stimulated L2/3 pyramidal cells were able to influence at least one neighboring unit significantly (“effective stimulators”) under anesthesia, compared to 5/14 (35.7%) in the awake state. When stimulating L4 neurons, we recorded 3/14 (21.4%) effective stimulators. **(B)** Left: Blue dots represent data from all L2/3 anesthetized recordings, red from L2/3 awake recordings, and green from L4 awake recordings. The average percentage of follower cells per recording is low regardless of layer and brain state (0.56–0.75%). Right: Same conventions but considering only data sets which had at least one significant follower. Error bars represent standard error of the mean. **(C)** Black dots: Mean z-scores and error bars (sem) of simulated datasets as a function of the probability of eliciting one AP per stimulation epoch. Blue Dot: Mean and sem of the mean z-score across the excited followers from all real stimulation experiments (including L2/3 and L4 stimulation), showing that on average, the statistical significance of identified follower cells is similar to cells with simulated excitation in 50% of trials. **(D)** Black dots: Mean (± sem) ΔF/F-values of “relevant” (trials that contribute to significance: see section Methods) trials of all simulated cells as a function of percent simulated trials. Blue Dot: Mean (± sem) ΔF/F-value of “relevant” trials across all excited followers including L2/3 and L4 stimulation. The ΔF/F response of excited followers was similar to simulated followers when an AP was added in 50% of their trials.

For validation and calibration of the new analysis algorithm, we used spontaneous activity recordings of patched cells containing OGB. We identified calcium transients corresponding to one AP in the patched cell, and superimposed them onto the Δ/F traces of multiple cells from the same recordings at artificial (mock) stimulus times. We repeated this process for different percentages of trials receiving extra APs, simulating different stimulation efficacies (see Figures 2C–E). We then proceeded to analyze the simulated activity the same way we analyzed actual stimulation recordings, thereby obtaining estimates for sensitivity thresholds and expected responses at certain stimulation efficacies (see Figures 2C–E,3C,D).

## Results

We stimulated 33 L2/3 pyramidal neurons, 19 in anesthetized and 14 in awake animals, and 14 L4 pyramidal neurons, all in awake animals. Most units were held in whole-cell configuration (18/19 L2/3 neurons in anesthetized & 11/14 in awake animals; 8/14 L4 neurons in awake animals), and the remaining in loose-patch configuration. Figure 1a (left) shows an example of a layer 2 pyramidal neuron filled with Fluoro-Ruby dextran, allowing us to visualize its apical dendritic tree and spines consistent with the pyramidal cell morphology (Figure 1b). In Figure 1a (right) we show an example of an L4 neuron, recorded in cell-attached mode (gray dashed circle), and the overlying L2/3 population, one layer of which was imaged. APs were elicited reliably (Figure 1c) and consistently over periods of up to 1 h (Figure 1d), and were typically evenly spread over the stimulus duration. Appropriate gaps (at least 2.5 s) between stimuli were given to allow calcium transients of potential follower cells to return to baseline before the next trial. The patched cell and the target population of neurons were labeled prior to patching with bulk-injected Oregon-Green BAPTA (OGB, Figure 1a; see section Methods), which allowed us to monitor the activity of individual units. OGB was used to ensure the dense labeling of the nearby cell population.

### The proposed method for identifying followers is sensitive and specific

As described in section Methods, we computed the response for each trial by averaging 400 ms of activity around the expected response peak (colored areas in Figure 2D). Figure 2A shows an example of our analysis for an excited follower (i), an inhibited follower (ii), and a neutral cell respectively (see section Methods and Figure 2A legend for a more detailed description). An important step to help interpret our results was to develop a model of our expectations, which depends on the characteristics of the OGB signal. Spontaneous activity recordings allowed us to quantify how OGB fluorescence measurements correspond to spikes in our hands: Using a custom template matching algorithm, single spikes were identified in the voltage trace of the patch recordings together with simultaneous calcium signal acquisition (Figure 1e). Corresponding calcium transients from the patched cell were extracted as described in section Methods, and the calcium response amplitude was plotted against the number of spikes in a firing event. As expected (Hofer et al., 2011; Nauhaus et al., 2011), the resulting relationship is predominantly linear, especially at low numbers of spikes, the range that is most relevant here (Figure 1f). This validation allows us to simulate the effect of added spikes on the calcium trace of a cell via linear summation.

Using spontaneous activity recordings from the cells we patched, we identified the typical OGB calcium transient that corresponds to an isolated, single recorded spike. We then simulated the signal that would be generated in a “follower” cell assuming a certain percentage of trials elicits one extra spike transient in the calcium trace of that cell. To do this we added single AP calcium transients to a variable percentage of ***mock*** (*simulated*) trials (Figure 2D), which were superimposed to the calcium trace of a cell firing spontaneously. Figure 2D shows the average calcium response when randomly chosen 1-AP transients were added to 10 (left), 40, 50, or 100% (right) of 190 simulated trials, which was the average number of trials used in the electrical stimulation experiments. Black traces represent controls, i.e., no added activity. Note that as the percentage of trials with one extra spike increases, the signals separate as expected.

We generated z-score distributions for the simulated stimulation traces corresponding to an extra spike generated in 10, 40, 50, and 100% of trials respectively, and the corresponding shuffled null distributions (see section Methods). We established conservative significance thresholds using the null distribution of z-scores derived from the sham stimulation (see section Methods; Figure 2B legend) and requiring that no followers are identified in any sham session (high specificity). Under these conditions, we found that simulating a single added spike in ~40% or more of the trials yields significant modulation. Naturally this threshold would further improve (decrease) if we simulate multiple spikes per trial, but simulating a single spike gives us a good sense of the limitations of our approach. It is also important to note that whether significance is reached or not depends on the variability of firing, which in turn depends on cell type and stimulation conditions. In practice, our estimate did not differ much from cell to cell in the spontaneously firing L2/3 pyramidal cell population whose traces we used for the simulations. We tested how our conclusions depend on the number of simulated trials and found similar results over a range from 100 to 400 trials, which covers the range of trials that realistically can be used for whole-cell patch electrical stimulation experiments. Finally, we note that comparisons based on the *t*-test, which typically compares calcium responses directly between stimulation and control conditions, are generally weaker in conferring statistical significance. For example, by *t*-test, there was no statistical significance between control and the simulated stimulation condition for which 1-AP transients were added in 40% of the trials. Figure 2C shows that the criteria we implemented allow the algorithm to be more sensitive for identifying followers than standard approaches (Kwan and Dan, 2012), while at the same time being specific enough to avoid detecting false positives in sham trials. By comparison, the (Kwan and Dan, 2012)-method, which deems a cell a follower if its mean stimulus response is > mean + 3 sem of the ΔF/F difference between pre- and post-stimulus epochs, is both less sensitive (Figure 2C) and less specific, yielding several false positive followers in our sham stimulation trials. Specifically, the number of followers identified in sham trials was ~40% of the number of followers identified in the real stimulation trials, with this method.

Note that we did not find any significant correlation between neuropil-ΔF/F calcium response amplitude and distance from the patched cell. This is reassuring, as it indicates that the calcium signal from the axon and dendritic processes of the patched cell did not significantly impose the activity generated from the stimulation upon the surrounding neuropil signal. Therefore, it is unlikely that follower cell activity was influenced directly by the fluorescent signal of the patched cell to a significant degree. Furthermore, the mean number of spikes elicited per epoch was high and stable (12–15 on average), and it would be unlikely that the fluctuations in firing rate caused the differences we observed (see Supplementary Results).

### Recruitment of follower cells is rare, irrespective of brain state

To compare recruitment of neighboring L2/3 neurons in the anesthetized vs. the awake state, 19 L2/3 pyramidal cells were patched and stimulated in 18 anesthetized animals while 14 additional L2/3 pyramidal cells were patched and stimulated in 11 awake animals. There were, on average, 47 ± 4.8 (sem) OGB-labeled cells per FOV in anesthetized and 64 ± 4.4 (sem) in awake experiments. We define cells that had at least one “follower” inside the FOV to be “effective stimulators” (Figure 3A). Four (21.1%) of the 19 cells patched in L2/3 under anesthesia were capable of influencing one or more surrounding neurons significantly when stimulated, within an average radius of 132 ± 71 μm (*SD*). In the awake state, there were 5 out of 14 (35.7%) “effective stimulators,” within an average radius of 173 ± 95 μm (*SD*), a difference that was not statistically significant (*p* = 0.35, chi-square test; see Figures 3A,B). Therefore, despite a general increase in inhibition in the awake cortex, as previously shown by Haider (Haider et al., 2013), the number of L2/3 neurons that are capable of recruiting neighbors does not depend strongly on brain state, as it does not seem to change much between quiet wakefulness and light anesthesia.

In order to ensure that differences in FOV sizes between anesthetized and awake experiments did not bias our results, we confirmed that our analysis yielded the same trend after standardizing FOV size, by excluding FOVs that were smaller than 150 μm in radius (*n* = 6 FOVs in anesthetized experiments, none in awake recordings), as well as cells that were >150 μm from the patched cell (within larger FOVs).

In L2/3 of anesthetized animals, on average 0.56% (±0.28 sem) of the cells per FOV had significantly modulated ΔF/F responses when we electrically stimulated a patched cell inside the FOV (Figure 3B). In awake experiments, this percentage was slightly higher at 0.75% (±0.35 sem, Figure 3B). When pooling the data from all FOVs together, only 0.5% (4/796) of all recorded neurons were identified as followers in anesthetized recordings. In the awake state, the corresponding percentage was 0.96% (8/832). These results include all the recorded sessions, including those from recordings where no followers were observed, and we did not include these values in the figure. Note that due to the rare nature of a cell being identified as a follower, statistical significance for the difference between anesthetized and awake recordings could not be achieved for these results. In L2/3 anesthetized experiments half of the followers were inhibited. In awake L2/3 stimulation experiments a quarter of the followers were inhibited, while in awake L4 stimulation experiments no inhibited followers were identified. However, statistically significant differences were not achieved due to the small number of followers.

In seven datasets we were able to separate all GABAergic from pyramidal neurons by red fluorescent labeling using the Viaat-Cre x Ai9 line (*n* = 247 pyramidal cells and 49 interneurons in total). Notably, none of the 3 follower cells we identified in these recordings were interneurons. In 22 separate datasets, we identified a total of 34 PV+ interneurons (using PV-Cre x Ai9 crosses). None of those were deemed followers, consistent with the findings of Kwan and Dan (Kwan and Dan, 2012). Eight additional datasets were from Dlx5/6-Cre x Ai9 crosses, expressing tdTomato in practically all PV+ and SOM+ interneurons. Out of the 32 identified interneurons in these recordings, none were followers. The 6 remaining mice had no labeling of interneurons (10 datasets, *n* = 276 cells), but we identified no followers in these recordings, so we can only infer that the interneurons we imaged in these recordings were also not recruited. Taken together, we did not find any prominent evidence of recruitment of interneurons through single pyramidal cell spiking within local cortical volumes in mouse V1.

### L4 awake stimulation is as weak as L2/3 stimulation in the awake state

We compared the ability of L2/3 cells to recruit neighboring units with the ability of stimulated L4 cells to recruit overlying L2/3 cells. Three out of 14 (21%) stimulated cells in L4 (awake) had followers inside a 250 × 250 μm field of overlying L2/3 neurons [avg. 56 ± 3.7 (sem) neurons per FOV, vs. 64 ± 4.4 (sem) in awake L2/3 stimulation recordings]. As in L2/3, the percentage of follower cells per recording did not depend on the number of cells imaged (slope = −0.06, *R*^2^ = 0.06) within the range of our experiments. The percentage of patched cells with at least one follower in L4 (21%) was not significantly smaller than in L2/3 awake experiments (35.7%; *p* = 0.35, chi-squared test). As in L2/3 awake experiments, when a patched cell was an effective stimulator it could only influence a small percentage of nearby L2/3 units. On average, 0.73% of cells per FOV were followers when an L4 cell was stimulated (4 followers out of 706 cells in total) vs. 0.75% for awake L2/3 cell stimulation. Notably, all L4 stimulation followers were excited followers in contrast to L2/3 stimulation experiments, which had both excited and inhibited followers. However, because the total number of followers was small, this difference was not statistically significant.

### How reliably do spikes get elicited in the followers?

Even though OGB has been shown to have reasonably good signal-to-noise-ratio for single AP detection (Kerr et al., 2005), there remains considerable variability in the calcium signal amplitude corresponding to single spikes (see Figure 1f). Therefore, it is not possible to determine whether a single stimulus epoch elicited an extra AP on a trial-by-trial basis. However, it is possible to estimate the percentage of effective trials by comparing the z-scores corresponding to the real follower against the z-scores obtained from simulated data (Figure 3C): On average, follower cells responded to stimuli in ~50% of all trials.

It is interesting to ask by how much the calcium signal of a follower increases per trial. The mean ΔF/F amplitude of a follower cell in response to all trials was 2.3% (±0.2 sem), shown as the blue dot with error bars in Figure 3D). This corresponds to the mean ΔF/F signal of all simulated followers when stimulated with one extra AP in 50% of all trials (2.2% ±0.1 sem). Since we estimate that most followers do get recruited in ~50% of all trials, it is likely that no more than 1 AP is elicited in each successful trial. We cannot rule out that some followers generated >1 additional AP per effective stimulus as a follower: producing multiple extra APs in a lower number of trials could yield a similar *z*-value distribution as one that received single extra APs in a higher number of trials. However, it is hard to imagine a scenario where a neuron firing the same number of spikes per epoch would drive a partner very strongly on rare epochs and not at all most of the time.

### Single neuron firing has subtle but significant influence on local ensemble activity

**Figure 4:**
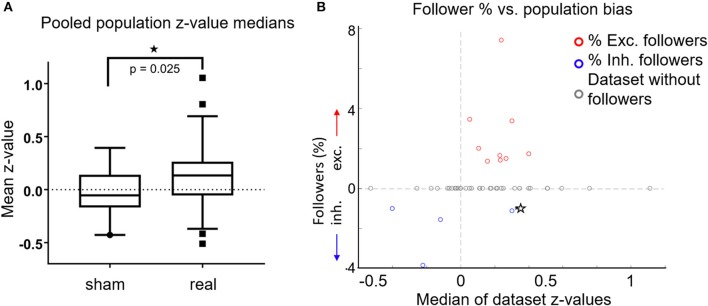
**(A)** For each FOV, we averaged the z-scores of all cells, for both sham experiments (*n* = 19) and real stimulation experiments (*n* = 47). Data from L2/3 anesthetized, L2/3 awake and L4 awake stimulation experiments were pooled together, because there were no significant differences between them. Box plots show the median (central horizontal line), 25th and 75th percentiles (upper and lower box boundary), and 5 and 95th percentiles (bottom, top whiskers, respectively). Medians were significantly different (*p* = 0.025, Wilcoxon ranksum test). **(B)** Percentage of followers per FOV as a function of the median z-score of all cells in each FOV. Red circles: percent of excited followers. Blue circles: percent inhibited followers. Gray Circles: datasets without followers. Star: A single dataset with both inhibited and excited followers. The population influence of a stimulated cell generally matched the type of followers present in the respective FOV.

We examined whether stimulation of a single neuron had any effect on the overall activity of all the cells in any given FOV. For each cell in an FOV, we used their mean *z*-value to calculate the population median (“z-median,” Figure 4A) for sham experiments (*n* = 19 FOVs, left box plot) vs. real stimulation experiments (*n* = 47 FOVs, right box plot) across all layers and brain states. We pooled the data since there were no significant differences observed across the three different stimulation categories. The z-median of the sham FOVs was −0.05, while the z-median corresponding to real stimulation data sets was higher at 0.135 (see Figure 4A; *p* = 0.025, Wilcoxon ranksum test). *Note that the z-median is not sensitive to the small number of relatively strong followers detected per FOV, but instead reflects the effect of single unit stimulation on aggregate population activity*. The result suggests that the activity of the stimulated cell does have, on average, a weak but significant excitatory influence, which is distributed across the local cell population. Interestingly, because it is generally weak, this influence manifests only rarely as significance at the single cell level, resulting in the observed small number of significant individual cell followers.

In Figure 4B we plot percent followers in each FOV as a function of the FOV's z-median. For visualization, inhibited follower percentages (blue circles) are plotted as negative values, excited followers as positive values (red circles). Gray circles denote datasets without followers, while the lone star indicates a single dataset (FOV) that had both excited and inhibited followers. Population activity in FOVs that had excited followers was generally elevated, and population activity in FOVs that had inhibited followers was generally suppressed. In other words, the population influence of a stimulated cell generally matched the type of followers present in the respective FOV, further demonstrating that stimulated single cells exert influence over their local neighborhood.

## Discussion

Despite remarkable progress in understanding neuronal properties made long ago (Hebb, 1949; Mountcastle, 1956; Hubel and Wiesel, 1962), little is known about how single units interact with other neurons to form functional groups, *in vivo*, within neocortical circuits. In the visual cortex, such sub-networks are thought to exist and be important for encoding and transmitting visual information efficiently (Yoshimura et al., 2005). It is therefore important to understand how neurons in these subnetworks influence each other. We define the “range of influence” of a single pyramidal neuron to be the downstream units whose output it can influence, directly or indirectly, when it fires. In principle, there may be different types of pyramidal cells: Some neurons might “prefer” spreading their activity out to many target neurons downstream, while others may “focus” their influence strongly on fewer, more specific, target cells, for example by having their axonal branch innervate multiple spines on the same postsynaptic dendrite (see Kasthuri et al., 2015). A recent study using multiple *in vivo* patch-clamp recordings reports a connectivity ratio of 6.7% between pyramidal cells, with more reliable connections displaying higher EPSP-values (Jouhanneau et al., 2015) In fact, it has been suggested that, on a spatial scale of 50–300 μm, neurons transiently organize themselves into functionally specific small-world networks in different cortical layers (Yoshimura et al., 2005; Kampa et al., 2006; Perin et al., 2011; Carillo-Reid et al., 2016). Therefore, it is possible that activating a neuron might elicit activity preferentially into members of its small-world network.

The impact that the firing of a single unit has for the entire nervous system, and indeed the organism itself, can be far reaching. Several studies (Brecht et al., 2004; Houweling and Brecht, 2008; Li et al., 2009; London et al., 2010; Ikegaya et al., 2013) suggest that the firing of one single unit can influence significantly both neighboring circuit activity and behavior. For example, London et al. (2010) estimate that a single extra spike in somatosensory cortex is responsible for ~28 additional spikes in its post-synaptic partners. Houweling and Brecht (Houweling and Brecht, 2008) argue that 14 extra APs occurring over 200 ms inside a single cell can be sensed by the mouse. (Brecht et al., 2004) show that in rat motor cortex, 10 APs from one intracellularly stimulated layer 5/6 pyramid firing at 50 Hz are sufficient to elicit single whisker movement, particularly in the awake state. Li (Li et al., 2009) shows that one high-frequency burst from a single cell can induce UP/DOWN network state transitions in rat somatosensory cortex. However, many of these inferences are indirect, and, moreover, they do not address how the efficacy of stimulation varies between layers or in different brain states.

Kwan and Dan (Kwan and Dan, 2012) recently reported that activating single L2/3 pyramidal cells in the anesthetized state elicits firing in ~1.7% of neighboring pyramidal cells, as well as a larger fraction (29%) of neighboring SOM+ cells. This study confirmed that network effects of single neuron activation, though weak, involve spike generation in nearby units. Here we go further to explore whether: (1) it is possible to establish how sensitive and robust the two-photon imaging calcium imaging technique is at identifying follower activity using a novel analysis algorithm and simulations of follower activity under anesthesia and awake conditions, both within L2/3 and from L4 to L2/3, and (2) whether we can also quantify more subtle yet distributed effects of single cell stimulation on the local activity beyond the rare, strong connections of reliable follower cells.

### A new statistical analysis approach with higher sensitivity for identifying followers

We expanded on Kwan and Dan's (Kwan and Dan, 2012) approach by introducing a more sensitive analysis algorithm for identifying follower cells. Our statistical analysis is based on comparing the effect of real stimulation epochs with null stimulation epochs, derived from each cell's own activity via circular shuffling (see section Methods). This approach leads to null distributions that take into account the noise and spontaneous variability of each cell, and has more power for detecting reliable followers without losing specificity. We assessed the sensitivity of this approach to correctly identify followers (Figure 2C), by testing it on data generated from spontaneous activity to which measured average calcium transients were added to mimic the effect of a single AP that is applied in a variable number of trials (Figure 2D). For a total number of trials commensurate to that obtained in our experiments, this approach correctly identified almost all follower cells for which stimulation elicited one extra spike in 60% of all trials (Figure 2C). Sensitivity dropped sharply after that, reaching ~50% chance of detection of followers in which stimulation elicited one extra spike in 40% of the trials (see also Figure 2E). The range of sensitivity to be identified as a follower can be seen in the width of the blue histograms in Figure 2E, and it predominantly stems from the unique firing and noise properties of each cell's recorded signals. Higher spike number generation per stimulation epoch would require fewer trials for detection. To ensure that no false positive followers were identified, we used sham stimulation recordings from 1,069 cells (incl. L2/3 anesthetized, awake and L4 awake recordings) as a benchmark to set conservative thresholds that ensure no sham followers (see section Methods, Figure 2B). Note that using the (Kwan and Dan, 2012) algorithm in our data yields 0.28% (3/1,069) false positive sham followers, as well as 0.31% real followers in our L2/3 anesthetized and awake stimulation experiments combined, vs. 0.74% with our algorithm. In this study, we specifically searched for reliable, extraordinarily strong connections between cortical neurons. Presumably, these connections are special, in that they may represent long-lasting structures for information processing and memory formation (Clopath et al., 2017). Followers with < 40% reliability may represent a different class of connections that are more transient in nature. Using different recording methods, such as extracellular multichannel recordings with silicon probes, may have allowed us to pick up followers responding to < 40% of stimulation trials, however, we would not have been unbiased to the type of cells we record from the way we are with two-photon calcium imaging. Additional studies will be required to address those in depth.

### Stimulation in quiet wakefulness vs. anesthesia

Lateral inhibition is enhanced in the awake state (Haider et al., 2013), suggesting that the range of influence of single neuron excitation may be reduced under these circumstances. However, we did not find a significant difference in the recruitment of reliable followers between the two states. Under anesthesia, 21% of patched L2/3 cells were capable of activating at least one other L2/3 neuron within the FOV examined compared to 36% in awake animals, but the difference was not significant (*p* = 0.35, chi-square test). It is possible that effective stimulators might have more followers beyond ~300 μm under anesthesia that were outside of the FOV sizes we used, and those connections may become weaker when the animal is awake. This hypothesis can be tested in future studies as more sophisticated imaging techniques with larger FOVs become available. In addition, the connections we identify between stimulator and follower, though infrequent, may be sufficiently stronger than the average cortex connectivity so that the effect of increased inhibition during wakefulness may not affect them enough to weaken the response of a follower to single-cell stimulation. Interestingly, even though our method of detecting followers is more sensitive than that of Kwan and Dan (Kwan and Dan, 2012), our results show even lower numbers of followers than they report: only 2 out of 796 cells (0.025%) in our L2/3 anesthetized recordings were significantly activated (2 more were significantly suppressed), which is lower than the 25/1169 (2.1%) followers reported by Kwan (see Figure 2C, *p* = 0.5e-3, chi-square test). When we pool all followers from all layers together (16/2,354 or 0.68%), this difference becomes even more significant (*p* = 0.2e-3, chi-square test). This difference, while significant, is in our opinion not entirely irreconcilable, as it may have to do with different imaging and scanning conditions, different optical noise or resolution properties of the microscope, differences in animal treatment and experimental timing, and differences in the mouse lines used.

We note that, within the range of FOVs we imaged, the fraction of followers did not systematically depend on the size of FOV, number of cells imaged or average distance from the stimulated cell (see section Results, page 21/22). Followers were identified over a range of distances spanning 23 to 350 μm from stimulated cells, but because of low numbers we could not determine whether they clustered around specific distances (Supplementary Figure 1).

#### Excited vs. inhibited followers

L2/3 stimulation yields two types of followers: those excited and those inhibited. Half of the observed followers were excited under anesthesia vs. 75% in the awake state. Interestingly, L4 stimulation (performed in the awake state) yielded only excited L2/3 followers. Although the numbers are small, and significance is not established, this suggests that effective stimulators in L4 may be preferentially involved in feedforward excitation circuits, while effective stimulators within L2/3 may drive interneurons as well as other pyramidal cells (Kwan and Dan, 2012; Xu et al., 2016). In rat prefrontal cortex, the majority of monosynaptic connections from pyramidal cells target GABAergic interneurons, however they are transient and dependent on behavior (Fujisawa et al., 2008). A recent study using optogenetic activation of pyramidal cells in L2/3 of somatosensory cortex found predominantly disynaptic inhibition (Mateo et al., 2011). However, they activated ~500 neurons at the same time, which may recruit disproportionally more inhibitory responses and lead to a different network response than our single-unit stimulation presented here.

Interestingly, using crosses of different Cre-driver mouse lines with the tdTomato-expressing Ai9 line, we found no preferential recruitment of the major classes of GABAergic interneurons (PV+, SOM+). In fact, we saw no interneurons recruited. However, it is also clear that some interneurons were activated to produce the inhibitory follower responses we found. Since many cortical interneuron subtypes are distributed quite sparsely and connect to large numbers of pyramidal cells (Jiang et al., 2015), it is quite possible that we did not see the ones we activated in the FOVs. We may be able to overcome this limitation in the future with 3-dimensional imaging techniques. There may also be translaminar inhibitory connections (Kapfer et al., 2007; Kätzel et al., 2011) that could have mediated disynaptic inhibition. Furthermore, it is possible that calcium imaging was not sensitive enough in certain subtypes of interneurons to detect these responses (single-AP transients in PV-positive interneurons were ~40% smaller than in pyramidal neurons, data not shown), or they might belong to a specific class of interneurons we did not have the ability to visualize. With more sensitive imaging techniques becoming available for future studies, it is possible that additional inhibitory follower cells may be detected.

While we focused in this study on the impact of pyramidal cell activity on the local circuit, it will also be important to map the effect of single-cell activity of GABAergic interneurons *in vivo*. Several studies indicate that inhibitory cortical connectivity is stronger in local circuits and may influence perception to a greater degree than pyramidal activity (Houweling and Brecht, 2008; Xu et al., 2016). Because inhibitory interneurons are more challenging to patch *in vivo* than pyramidal cells, we anticipate this line of research to provide important new insights as newer techniques, such as SLM (spatial light modulator)—guided optogenetic stimulation of specific neuronal cell types becomes more widely available.

### L4 pyramidal neurons are approximately as effective at recruiting L2/3 activity

Previous studies have shown that interlaminar excitatory connectivity between L4 and L2/3 is stronger than intralaminar connectivity within L2/3 (Xu et al., 2016), while inhibitory connections unto pyramids are similar within L2/3 as from L4 to L2/3. This had us speculating whether we would see higher fractions of followers in L2/3 when we stimulated L4 pyramids. Surprisingly, we did not find a significant difference in the number of effective stimulators in L4 vs. L2/3, nor in the percentage of followers per FOV or per effective stimulator. One possible explanation is that we probed these connections *in vivo* where all long-range connections are intact, while the previously mentioned study used slice preparations and glutamate-photostimulation targeting multiple neurons within ~100 μm (Xu et al., 2016), so the recording and stimulation methods were quite different. The excitatory connectivity from L4 to L2/3 they report also increased with depth (see Figure 4 in Xu et al., 2016), and the L4 cells we stimulated were located in the upper half of L4, where they show similar EPSC amplitude and number as with L2/3 to L2/3 connections. We did not find any inhibited followers after L4 stimulation, so it is possible that strong connections to L2/3 neurons are mostly made up of direct excitatory inputs as opposed to the mixture of excitatory and inhibitory responses we saw from intralaminar L2/3 stimulation (Xu et al., 2016). However, there is no statistical support for these speculations due to the surprisingly low number of followers we found.

### Strength of elicited responses

The activity in excited follower cells is increased only by a small amount as a result of stimulating electrically a neighboring neuron (Figure 3D). This is true in anesthetized and awake recordings alike and approximately corresponds to 1 additional spike elicited per stimulation trial for a fraction of trials. We estimate that, on average, reliable followers appear to fire an extra AP in ~40–50% of stimulated trials (Figure 3C), the mean ΔF/F response over all trials being ~2.3% (Figures 2Ai,3D). Similar changes in ΔF/F were observed in the opposite direction in inhibited followers (Figure 2Aii). Similar to our estimate, (Kwan and Dan, 2012) found that all pyramidal followers responded to the stimuli of the patched cell only in a subset of all trials, whereas SOM+ interneuron followers (30% of all SOM+ interneurons they recorded) responded to nearly every stimulation trial. We never found any followers (out of >2,400 neurons) with *z*-values or mean ΔF/F responses corresponding to 100% of all trials, even though we should in principle have recorded from ~75 SOM+ neurons (in anesthetized and awake L2/3 stimulation recordings combined), because ~20% of all cortical neurons express GABA, out of which ~23% are SOM-positive in L2/3 of mouse V1 (Markram et al., 2004; Gonchar et al., 2008). One possible reason is that (Kwan and Dan, 2012) might have targeted Pyramid-SOM+ cell pairs that were near each other inside the imaging plane.

### Follower recruitment does not depend strongly on the local activity state

In addition to differences between global brain states (i.e., awake vs. anesthetized), we asked if local activity states had an effect on follower recruitment by analyzing the membrane potential (V_m_) from whole-cell patch recordings to interrogate the role of synaptic activity levels in the local cortical vicinity. Subthreshold V_m_ correlates strongly with the local field potential (Haider et al., 2006; Poulet and Petersen, 2008), which originates from the aggregate synaptic potentials of the local neighborhood (Legatt et al., 1980). Thus, we computed the mean V_m_ during the 100 ms leading up to each stimulus and computed its linear correlation with the follower cells' ΔF/F responses from the same recordings. We did not find any significant correlations. We also compared the distributions of V_m_-values before relevant and irrelevant trials, and did not find any difference. In addition, the baseline ΔF/F activity level (mean of 400–0 ms before stimulus onset) of follower cells did not predict the success or failure of individual stimulation epochs. This suggests that, within the parameters of our experiments, the recruitment observed here did not depend strongly on the moment-to-moment fluctuations of activity levels in the local cortical network. It is possible that the subgroup of neurons identified here may be biased toward cells that are more strongly connected and therefore less dependent on underlying brain “inner state” activity. Alternatively, it may be that there is an active process adjusting for internal state fluctuations in order to leave functional connectivity across cortical cells invariant.

### Single cell stimulation effects are distributed across the L2/3 population

Interestingly, we observed a significant shift in population z-score medians (“z-medians”), a measure of how much population activity changes as a result of single-cell stimulation, between FOVs receiving real (*n* = 47) compared to sham stimulation (*n* = 19). Figure 4A shows that population z-medians derived from real stimulation (pooling results from all layers) are shifted toward excitation by ~0.16 (*p* = 0.025, Wilcoxon ranksum test) compared to sham stimulation. This is below the threshold required for any single cell to be identified as a reliable follower (Figure 2B), but does indicate that single pyramidal cell activation has effects that are more widely distributed, and can raise the firing probability in more than a few of its target neurons.

Note that the z-scores for most of the followers we detected were consistent with their firing one extra AP in 40–50% of their trials (Figures 3C,D). For this type of follower, the sensitivity of our detection method is ~40% (Figure 2C), so it is possible we may be underestimating the number of true followers by a factor of two. In fact, it is important to realize that we may be missing a much larger number of potential followers, whose stochastic responses to the stimulated cell average one AP in <30% of the stimulated trials. The fact that we can detect a significant difference in the aggregate population response upon single cell stimulation suggests that this is indeed the case. This observation underscores the weak but distributed nature of the impact that single cell firing has on neighboring microcircuit responses. As more sophisticated and sensitive imaging and stimulation techniques emerge, it should become possible to quantify in greater detail to what degree certain cells are recruited more or less strongly by single-unit firing, and whether their coupling strength depends on other cellular characteristics.

## Conclusion

The observation that only very few neurons have reliable followers while influencing most other units weakly agrees with a recent study that found highly skewed distributions of synaptic connectivity, with 7% of most correlated pairs accounting for ~50% of the total synaptic weight in V1 (Cossell et al., 2015). Our findings qualitatively fit a connectivity model suggesting that cells weakly connect to most of their targets except for a minority of relatively strongly connected partners. This arrangement may favor the formation of neighborhoods or “cliques” of units, postulated to be important for processing sensory stimuli in the cortex (Cossell et al., 2015). Future studies will be able to address these issues more comprehensively with techniques such as optogenetic single-cell stimulation using light-sculpting methods (e.g., spatial light modulation), which have recently matured to the required level of precision (Packer et al., 2015).

## Code availability

The source code for all custom MATLAB routines can be made available to readers upon request to the corresponding author.

## Ethics statement

All experiments and animal procedures were performed in accordance with guidelines of the National Institutes of Health for the care and use of laboratory animals and were approved by the IACUC at Baylor College of Medicine.

## Author contributions

JM: performed experiments, analyzed data, and wrote the manuscript; PG: supervised experiments and edited the manuscript; SS: conceived the project and wrote the manuscript.

### Conflict of interest statement

The authors declare that the research was conducted in the absence of any commercial or financial relationships that could be construed as a potential conflict of interest.
