# Supplementary material for: The Effect of Single Pyramidal Neuron Firing Within Layer 2/3 and Layer 4 in Mouse V1
Source: Front Neural Circuits. 2018 Apr 16;12:29. doi: 10.3389/fncir.2018.00029 (PMC5911487; doi:10.3389/fncir.2018.00029)
Supplement: Supplementary file 1 [file DataSheet1.docx]

**Supplemental results**

There was no dependence between the average number of elicited spikes and the percentage of follower cells for each recording (L2/3 anesthetized: linear regression slope < 0.01, R^2^ < 0.01; L2/3 awake: slope = -0.1, R^2^ = 0.02; L4 awake: slope = 0.02, R^2^ < 0.01). The mean number of spikes/stimulus elicited per recording did not differ between the three experimental conditions (L2/3 anesthetized: 15.3 ± 2.9 (mean ± sem), L2/3 awake: 12.7 ± 1.1, L4 awake: 11.9 ± 1.7, p > 0.99, Kruskal-Wallis test with multiple comparisons correction). For comparison, (Kwan et al. 2012) found that stimuli >5AP were strong enough to recruit follower cells, so our stimuli were essentially always above that threshold.

FOVs in awake recordings were, on average, larger than in anesthetized recordings to contain more cells. In L2/3 stimulation recordings, the average distance of the imaged cells from the patched cell was 132 ± 71 µm (SD) in anesthetized animals, and 173 µm ± 95 in awake recordings. However, there was no correlation between the fraction of follower cells and the average distance of ROIs from the stimulated cell per FOV. It is therefore very unlikely that smaller FOVs would bias the fraction of followers per experiment towards higher values simply because of shorter distances. To ensure that is the case, we also analyzed the data omitting smaller FOVs that did not reach at least 150 µm, as well as all cells from larger FOVs that were >150 µm from the patched cell (see results section). FOVs from L4 awake stimulation experiments had an average diameter of 251 ± 12 µm (SD) containing 56 ± 12.6 (mean ± SD) cells, and L2/3 awake FOVs were on average 284 ± 41 µm in diameter with 65 ± 18 cells. There was no correlation between FOV diameter and follower percentages in L4 awake stimulations (R^2^ = 0.1), as well as in L2/3 awake stimulation experiments (R^2^ = 0.01).

The mean of the z-scores across all excited followers was 2.3 (±0.17 sem) for L2/3 anesthetized recordings, 2.55 (±0.3 sem) for L2/3 awake recordings and 2.3 (±0.09 sem) for L4 awake recordings. Pooling data from L2/3 anesthetized, awake and L4 awake trials together yielded a mean z-value of 2.42 (±0.15 sem, blue dot and error bars in fig. 3c). This was very close to the mean z-value obtained when we simulated an extra elicited AP in 40-50% of all trials (see fig. 3c, 2e), indicating that on average, a real, excited follower in our experiments was successfully stimulated in 40-50% of all trials. The range of mean z-values for all excited followers was [2.07 to 3.98], translating approximately to a single extra AP generated with probability 30% to 90% respectively per stimulation epoch, based on our simulation.

Figure S1 shows histograms of the distances of all non-follower cells from all L2/3 anesthetized recordings (left, black bars), as well as from all L2/3 awake recordings (right, grey bars). Superimposed are the locations of all excited followers (red lines) and inhibited followers (blue lines). Followers do not seem to cluster at particular distances around the stimulated cell, but due to their low number, it was not possible to be confident of this statistically.

Figure S2 summarizes the statistical process of identifying neurons as follower cells. See the methods section for details pertaining to this visualization of the analysis algorithm.


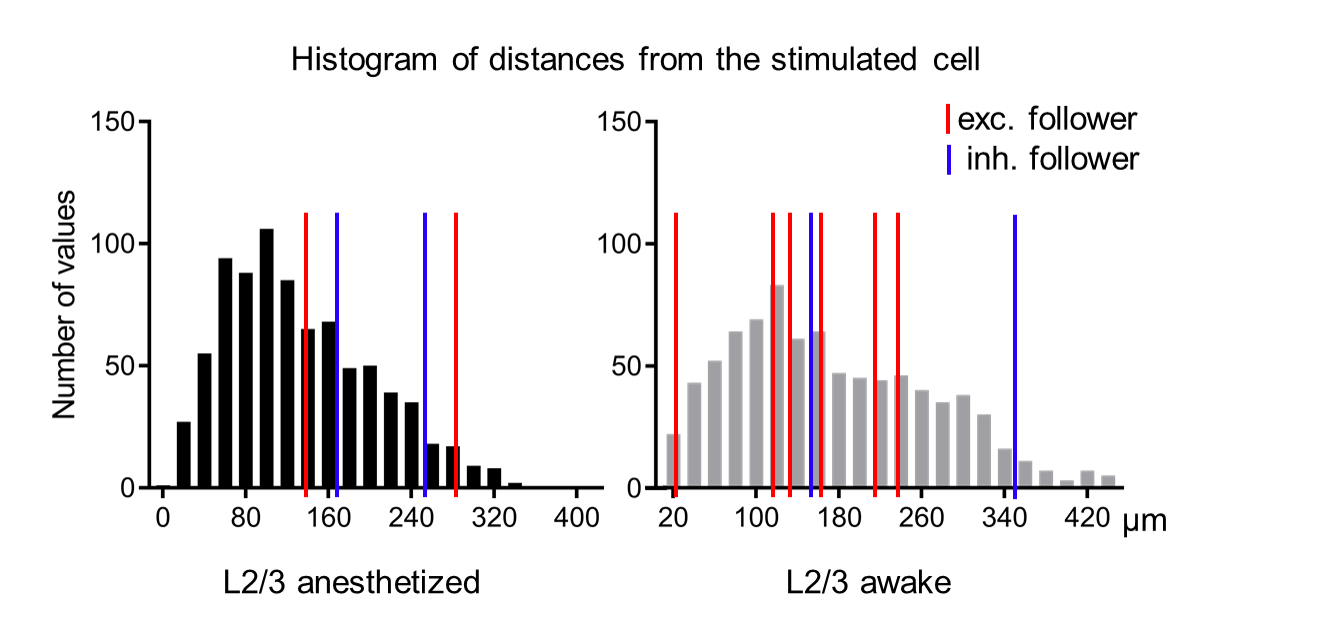


Fig. S1: Histogram of distances of cells in the FOV from the stimulated cell. *Black bars*: L2/3 anesthetized recordings. *Grey bars*: L2/3 awake recordings. *Red lines*: positions of excited followers. *Blue lines:* positions of inhibited followers.


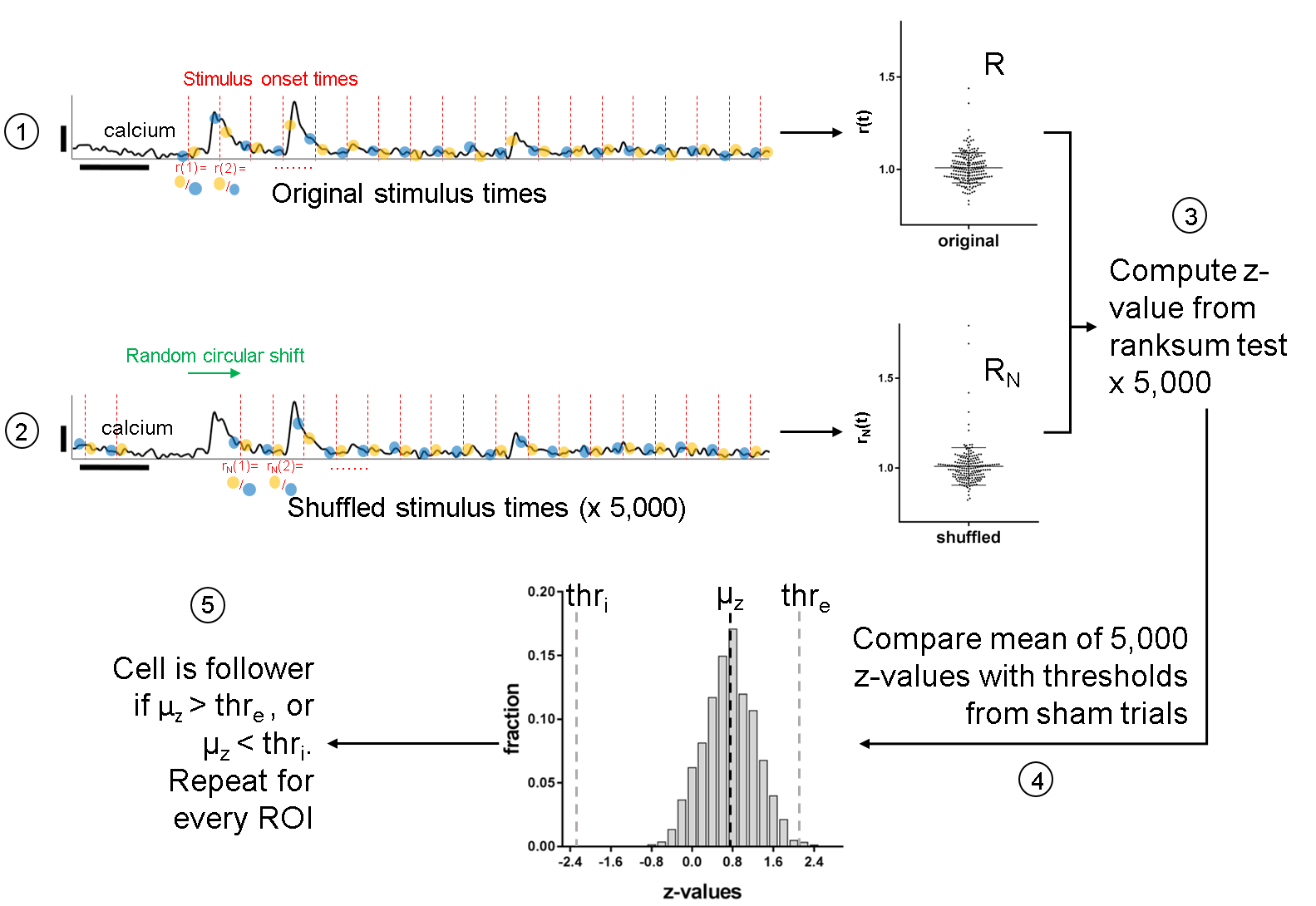


Fig. S2: Workflow diagram outlining the process of computing whether a cell was a stimulation follower or not. 1: The fluorescence trace after filtering out low-frequency drift. Stimulus responses r_x_ are calculated by dividing the average of 400 ms of activity (yellow circles) centered around the peak of the response, by 600 ms average activity preceeding the stimulus onsets (red dashed lines), yielding a distribution containing as many data points as there were stimuli. Vertical scale bar: 50% ΔF/F, horizontal scale bar: 10 sec. 2: The same process is performed 5,000 times for circular permuted stimulus times, creating 5,000 null distributions. 3: 5,000 ranksum tests are run between the original distribution R of r(t) values and the distributions R_N_ of r_N_(t) values. 4: The mean of the resulting 5,000 z-values, µ_z_, is compared with two thresholds derived from sham stimulations, thr_e_ (2.1) for excited followers, and thr_i_ (-2.3) for inhibited followers. 5: If µ_z_ is greater than thr_e_, then the cell was an excited follower, if it was smaller than thr_i_, it was an inhibited follower. The example shown here has µ_z_ falling within the thr_i_ to thr_e_ range and is not a follower.

| **Supplementary Table S1** | Kwan, et al., 2012 | This study |
| --- | --- | --- |
| Followers, Layer 2/3 -> Layer 2/3 anesthetized, target all neurons | 2.10% | 0.50% |
| Followers, Layer 2/3 -> Layer 2/3 anesthetized, target pyramidal neurons only | 1.7% (n = 1,152, "putative pyramidal") | 1.2% (n = 247, from 6 animals) |
| Followers, Layer 2/3 -> Layer 2/3 anesthetized, target PV+ interneurons only | 0% (n = 20) | 0% (n = 34) |
| Followers, Layer 2/3 -> Layer 2/3 anesthetized, target SOM+ interneurons only | 29% (n = 17) | N/A |
| Followers, Layer 2/3 -> Layer 2/3 anesthetized, target Dlx5/6 interneurons only (PV+ and SOM+ interneurons) | N/A | 0% (n = 32) |
| Followers, Layer 2/3 -> Layer 2/3 anesthetized, target all GABAergic interneurons only | N/A | 0% (n = 49) |
| Followers, Layer 2/3 -> Layer 2/3 awake, target all neurons | N/A | 0.96% |
| Followers, Layer 4 -> Layer 2/3 awake, target all neurons | N/A | 0.57% |
| Number of patched neurons, L2/3 anesthetized (out of those had followers) | 26 (N/A) | 19 (4) |
| Number of patched neurons, L2/3 awake (out of those had followers) | N/A | 14 (5) |
| Number of patched neurons, L4 awake (out of those had followers) | N/A | 14 (3) |
| Number of imaged neurons, L2/3 anesthetized (animals) | 1,189 (22) | 796 (18) |
| Number of imaged neurons, L2/3 awake (animals) | N/A | 832 (11) |
| Number of imaged neurons, L4 awake (animals) | N/A | 706 (10) |
